# Superoxide dismutase alterations in COVID-19: implications for disease severity and mortality prediction in the context of omicron variant infection

**DOI:** 10.3389/fimmu.2024.1362102

**Published:** 2024-02-22

**Authors:** Jinshen Chu, Lin Hua, Xiaofeng Liu, Huomei Xiong, Fangtinghui Jiang, Wei Zhou, Lu Wang, Guohui Xue

**Affiliations:** ^1^ Department of Clinical Laboratory, Jiujiang No.1 People’s Hospital, Jiujiang, China; ^2^ Department of Microbiology, Jiujiang Center for Disease Control and Prevention, Jiujiang, China

**Keywords:** superoxide dismutase, omicron variant, disease severity, biomarker, COVID-19

## Abstract

**Background:**

In the few reports to date, the changes in superoxide dismutase (SOD), a key factor in cellular protection against superoxide, in COVID-19 have been very inconsistent and contradictory. There is also a lack of data on COVID-19 induced by Omicron variant. Further investigation is warranted to figure out SOD alterations in COVID-19, particularly within the context of ongoing Omicron variant infection, which may provide clues to its role within COVID-19 pathogenesis and open up new avenues for COVID-19 treatment.

**Methods:**

SOD activity in 109 COVID-19 patients (including 46 severe cases and 63 mild to moderate cases) and 30 matched healthy controls were quantified. Demographic data, blood cell counts, biochemical indicators, coagulation indicators, and inflammatory markers were also recorded.

**Results:**

SOD, an important key node, experienced a significant decrease in COVID-19, with the severe patients exhibiting lower activity compared to the mild to moderate patients and control healthy. Notably, severe patients who deceased had the lowest SOD activity. Correlation analysis revealed significant correlations between SOD and inflammatory markers, organ injury markers, coagulation dysfunction indicators, nutritional markers, and lymphocytes counts. The ROC curve also showed good performance for the differentiation of severe cases and the prediction of death.

**Conclusion:**

SOD activity was significantly decreased in COVID-19 infected with Omicron variant and significantly correlated with systemic changes, and could be used as a biomarker to assess disease severity and predict mortality in COVID-19 clinical pathway management. Additionally, this finding will contribute to exploring new potential direction for the treatment of severe COVID-19 patients.

## Introduction

Severe acute respiratory syndrome coronavirus 2 (SARS-CoV-2), the causative agent of Coronavirus Disease 2019 (COVID-19), has rapidly spread across the globe, resulting in a significant public health crisis. Since its initial identification in Wuhan, the COVID-19 virus has undergone several mutations, including the Alpha, Beta, Gamma, Delta, and Omicron variants. Despite a decrease in the widespread nature of the COVID-19 epidemic, hundreds of thousands confirmed cases are still being reported daily around the world. In early 2023, China experienced a wave of COVID-19 outbreaks, with the Omicron variant as the predominant strain (1). This variant has over 50 mutations in the spike protein, which may increase its transmissibility and resistance to antibodies. Although there are indications of reduced clinical severity, elderly individuals with underlying health conditions are at risk of developing respiratory distress and multi-organ failure, which leads to high morbidity and mortality rates. As the world continues to grapple with the ongoing COVID-19 pandemic, researchers are tirelessly working to identify simple, easily detectable, and accurate biomarkers that are critical for primary hospitals in predicting severe cases and reduce morbidity and mortality rates.

Oxidative stress, characterized by an imbalance between reactive oxygen species (ROS), is a fundamental physiological condition of a healthy organism (2). However, when faced with pathogen invasion, the host organism activates pro-oxidative enzymes, which produce large amounts of ROS (3). ROS are signal molecules that lead to the production of pro-inflammatory cytokines and are related to the activation of T cell and B cell subsets (4). Additionally, dysregulation of oxidative stress and neutrophil recruitment plays a crucial role in the pathogenesis of acute respiratory distress syndrome, a characteristic of severe COVID-19 (5). Previous study have established a correlation between the involvement of ROS in the pathogenesis of respiratory viruses including SARS-CoV-2 (6). Superoxide dismutase (SOD), an important antioxidant enzyme, plays a critical role in scavenging superoxide radicals and participates in the signaling pathways of reactive oxygen species in microorganisms themselves, particularly in the case of eukaryotic pathogens (7). Immunohistochemical analyses of the inflammatory vital organs of patients who died from COVID-19 have shown increased levels of mitochondrial superoxide dismutase (SOD2) in the vital organs, but reduced levels in the lungs (8). Increase in plasma SOD activity and higher serum concentrations of SOD have been observed in two studies (9, 10). Moreover, A study analyzed changes in SOD activity in male and female patients with moderate COVID-19 of different ages (11). However, the results of other reports are inconsistent and inconclusive (12, 13), underscoring the need for a systematic evaluation of the available evidence. Although these studies primarily focused on COVID-19 induced by non-Omicron variants, the fundamental pathways of COVID-19 pathogenesis remain largely consistent across various SARS-CoV-2 variants. Therefore, we propose the hypothesis that SOD is also implicated in the pathogenesis of COVID-19 induced by the Omicron variant. In this paper, we aim to examine changes in serum SOD levels in COVID-19 patients infected with Omicron variant and specifically investigate the relationship between SOD and the function of various systems and illness severity. This information may provide insights into the pathophysiology of COVID-19 and inform the development of novel therapeutic approaches to improve patient outcomes.

## Materials and methods

### Study population

This study enrolled 109 patients newly admitted to Jiujiang No.1 People’s Hospital from November 2022 to February 2023. The Omicron variant was identified through whole genome sequencing at the laboratory of Jiujiang Center for Disease Control and Prevention. All cases included had complete medical records and laboratory test data, and tested positive for SARS-CoV-2. Patients who did not meet the above criteria were excluded from the study. The enrolled subjects were classified into two groups: mild to moderate (n=63) and severe (n=46), based on the criteria for severe and critical cases outlined in the Diagnosis and Treatment Protocol for SARS-CoV-2 Infection (Trial Version 7) in China. In addition, a control group of 30 healthy participants who were matched for age, sex and COVID-19 vaccination status from medical examination center during the same period was selected. This study received approval from Ethics Committee of Jiujiang NO.1 People’s Hospital (registration number, JJSDYRMYY-YXLL-2022-014), and confidentiality rights of individuals were upheld in accordance with the Helsinki Declaration.

### Data collection and laboratory analysis

All patients enrolled in this study were assigned unique identification numbers. Comprehensive medical records were collected, encompassing demographic information such as age, gender, height, weight, heart rate, admission systolic and diastolic blood pressure, as well as data on any existing comorbidities. Corresponding venous blood samples were obtained within 48 hours of hospital admission using the appropriate blood collection tubes. The analysis of these blood samples was performed using state-of-the-art laboratory instrumentation. Specifically, the Simens Advia 2400 automated biochemical analyzer was employed to measure a range of biomarkers, including superoxide dismutase (SOD), alanine aminotransferase (ALT), aspartate aminotransferase (AST), alkaline phosphatase (ALP), gamma-glutamyl transferase (GGT), total protein (TP), prealbumin (PA), albumin (ALB), blood urea nitrogen (BUN), creatinine (CR), uric acid (UA), triglycerides (TG), total cholesterol (TC), apolipoprotein A (APOA), apolipoprotein B (APOB), high-density lipoprotein (HDL), low-density lipoprotein (LDL), creatine kinase-MB (CKMB), lactate dehydrogenase (LDH), and hydroxybutyrate dehydrogenase (HBDH). Furthermore, the IMMAGE 800 system, based on immunoturbidimetric assay, was employed to quantify high-sensitivity C-reactive protein (hs-CRP), while the Snibe MAGLUMI X8 chemiluminescence platform was utilized for the measurement of interleukin-6 (IL-6). For hematological parameters, the Sysmex XN2000-A1 analyzer was utilized to determine white blood cells (WBC), red blood cells (RBC), hemoglobin (HGB), mean corpuscular volume (MCV), red cell distribution width-coefficient of variation (RDW-cv), platelets (PLT), mean platelet volume (MPV), platelet distribution width (PDW), neutrophils (NEU), lymphocytes (LYM), and monocytes (MON). Additionally, the measurement of lymphocyte subpopulations counts was carried out using the Beckman Coulter DxFLEX flow cytometer. Finally, the Sysmex CA-7000 automated coagulation analyzer was utilized to assess prothrombin time (PT), activated partial thromboplastin time (APTT), fibrinogen (FBG), thrombin time (TT), D-Dimer, fibrin degradation product (PFDP), and antithrombin III (AT3). The reagents used for serum SOD detection were purchased from MedicalSystem Biotechnology CO.,LTD (China), with the product code H439A.

### Statistical analysis

The data statistical analyses were performed using SPSS version 23.0 and GraphPad Prism version 8.0.2 software packages. The normality of the data distribution was assessed using the Shapiro-Wilk test. For normally distributed continuous variables, the independent t-test was employed to compare means. In cases where the data did not follow a normal distribution, the Mann-Whitney U test was utilized. Categorical variables were compared using the chi-square test and expressed as percentages. Spearman’s rank correlation analysis was employed to examine the relationships between variables. The diagnostic performance of the disease indicators was assessed by constructing receiver operating characteristic (ROC) curves. Statistical significance was indicated as *p<0.05, **p<0.01, and ***p<0.001, using a two-tailed p-value of 0.05.

## Results

### Subjects characteristics

A total of 109 COVID-19 patients were enrolled in this study, including 60 males (55.0%) and 49 females (45.0%), with ages ranging from 22 to 96 years. Of these, 63 patients had mild symptoms, ranging in age from 29 to 96 years, including 27 males (42.9%) and 36 females (57.1%). Meanwhile, 46 patients had severe symptoms, including 33 men (71.7%) and 13 women (28.3%), aged 22 to 89 years. There were 30 subjects in the control group, including 12 men (40.0%) and 18 women (60.0%), aged 36 to 87 years. No significant differences in age, sex, BMI or COVID-19 vaccination status were found between the COVID-19 and healthy controls (**Table 1**), nor between mild to moderate cases and severe patients. Heart rate was significantly higher in the severe group. However, no significant differences were observed in systolic and diastolic blood pressure at baseline or in major comorbidities such as diabetes, hypertension, vasculitis, cardiovascular disease (CVD), cancer, chronic kidney disease (CKD), COPD and uremia (see **Table 2**).

**Table 1:** Demographic data characteristics of all subjects.

	COVID-19 (n=109)	Control (n=30)	p value
Age, y, median(IQR)	74 (63.5-83)	69 (65-76)	0.114
Male, n(%)	60 (55.0)	12 (40.0)	0.144
BMI (kg/m^2^)	23.61 (20.785-26.57)	24.91 (22.24-26.67)	0.430
Vaccination status (yes), n(%)	105 (96.33)	29 (96.67)	0.930

**Table 2:** Clinical characteristics of COVID-19 patients.

	Total (n=109)	mild to moderate (n=63)	severe (n=46)	p value
age, y, median (IQR)	74.00 (63.50-83.00)	73.00 (61.00-81.00)	75.00 (66.50-83.00)	0.236
male, n (%)	60 (55.00)	27 (42.90)	33 (71.70)	0.144
BMI (kg/m^2^)	23.61 (20.79-26.57)	23.61(20.66-26.29)	24.03 (20.76-26.83)	0.680
heart rate (/min)	82.00 (77.00-92.00)	79.00 (72.00-86.50)	89.50 (78.00-105.50)	*<0.001*
SBP (mmHg)	129.00 (113.25-141.75)	130 (116.00-142.25)	128 (110.00-140.75)	0.515
DBP (mmHg)	76.00 (70.00-82.00)	75.00 (70.00-82.00)	76.00 (67.75-82.00)	0.760
main comorbidities				
diabetes, n (%)	25 (23.00)	13 (20.63)	12 (26.09)	0.504
hypertension, n (%)	43 (39.50)	21 (33.33)	22 (47.83)	0.128
vasculitis, n (%)	2 (1.80)	0(0.00)	2 (4.35)	0.095
CVD, n (%)	10 (9.20)	7 (11.11)	3 (6.52)	0.412
cancer, n (%)	7 (6.40)	4 (6.35)	3 (6.52)	0.971
CKD, n (%)	1 (0.90)	1 (1.59)	0 (0.00)	0.391
COPD, n(%)	4 (3.70)	4 (6.35)	0 (0.00)	0.082
uremia, n (%)	1 (0.90)	0 (0.00)	1 (2.17)	0.240

BMI, body mass index; COPD, chronic obstructive pulmonary disease; CVD, cardiovascular disease; CKD, chronic kidney disease.

### Serum SOD in COVID-19

**Figure 1:**
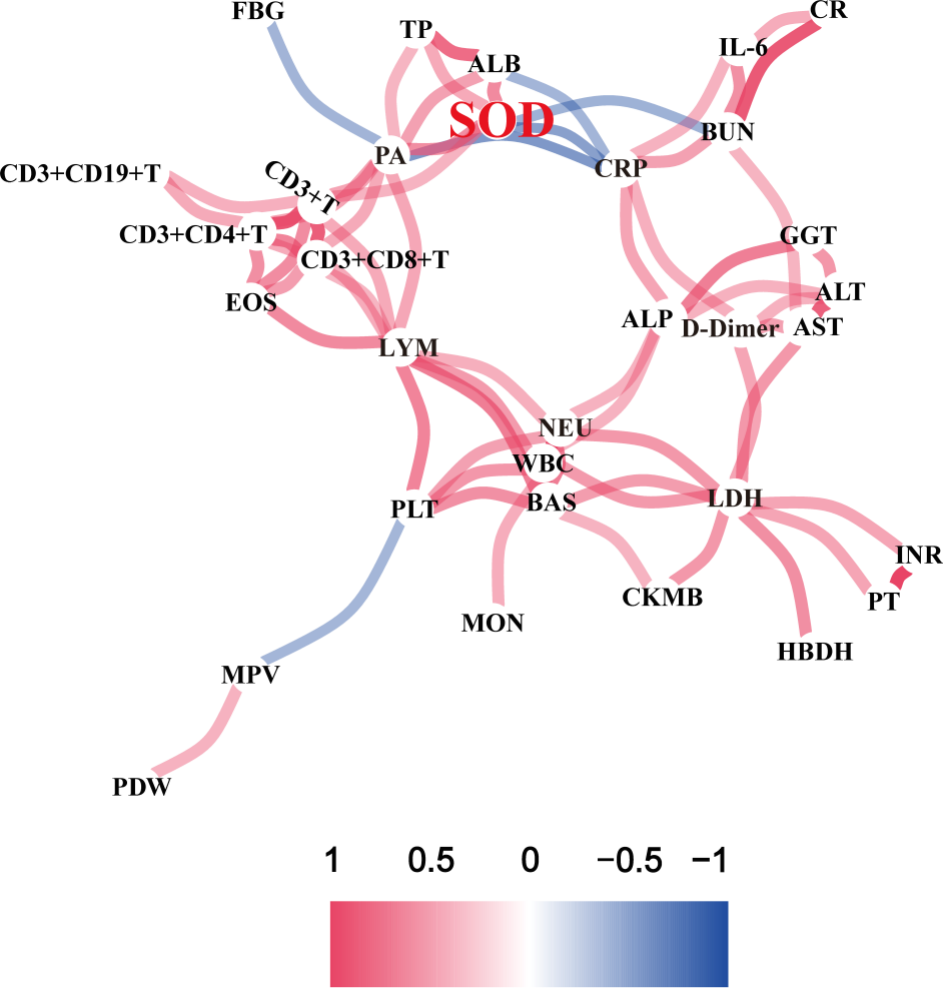
Correlation network diagram of various indexes in COVID-19 patients.

**Figure 2:**
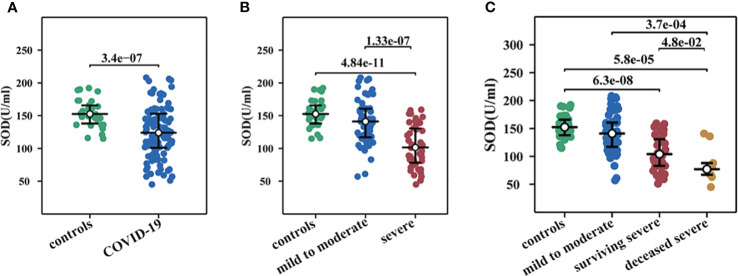
Changes of serum SOD activity in COVID-19. **(A)** SOD in COVID-19 and healthy controls. **(B)** SOD in mild to moderate COVID-19, severe COVID-19, and healthy controls. **(C)** SOD in healthy controls, mild to moderate COVID-19, survival severe COVID-19, and deceased severe COVID-19.

In order to explore the changes of different infection traces and the interconnection between different indicators, we drew a correlation network diagram (**Figure 1**) and found that SOD is one of the important key nodes, which is linked with nutritional indicators such as TP, ALB, and PA, inflammation indicators such as CRP, and organ damage indicator BUN. This suggested SOD may be a broad-spectrum composite indicator of the systemic status in a multidimensional manner. Statistical analysis of SOD changes in different groups were performed and a significant decrease was observed in COVID-19 (**Figure 2A**). After categorizing COVID-19 group into mild to moderate and severe groups, we observed severe subjects had lower SOD levels compared to non-severe patients and healthy controls (**Figure 2B**). Notably, we found that the lowest decrease in SOD occurred in severe deceased patients (**Figure 2C**).

### Correlations between SOD and inflammation markers

**Figure 3:**
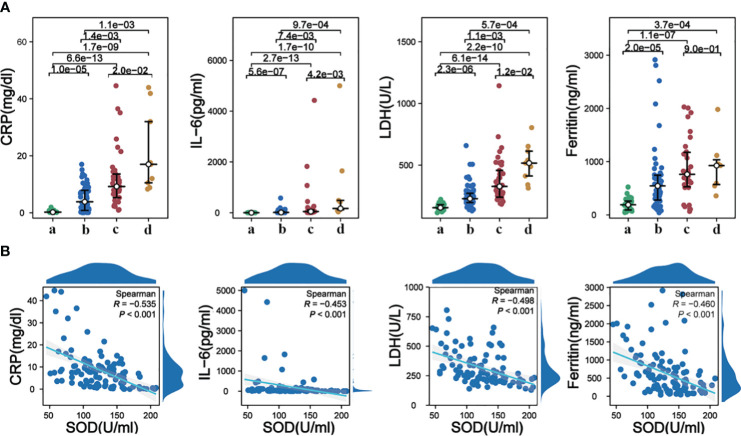
**(A)** Alterations in serum levels of CRP, IL-6, LDH, and Ferritin among different groups; **(B)** Correlations analysis of serum SOD with CRP, IL-6, LDH, and Ferritin. a, healthy controls; b, mild to moderate COVID-19; c, survival severe COVID-19; d, deceased severe COVID-19.

Inflammatory markers, namely CRP, IL-6, LDH, and Ferritin, exhibited a significant elevation in COVID-19 cases as compared to the control group. It was further noted that these inflammatory markers displayed higher levels in deceased individuals with severe cases, as opposed to surviving severe subjects, with the exception of Ferritin (**Figure 3A**). Notably, correlation analysis revealed a significant negative association between SOD and CRP (r = -0.535, p < 0.001), IL-6 (r = -0.453, p < 0.001), LDH (r = -0.498, p < 0.001), and Ferritin (r = -0.460, p < 0.001) (**Figure 3B**), suggesting a direct connection between oxidative stress and the inflammatory response in COVID-19.

### Correlation of SOD with immunocytes

**Figure 4:**
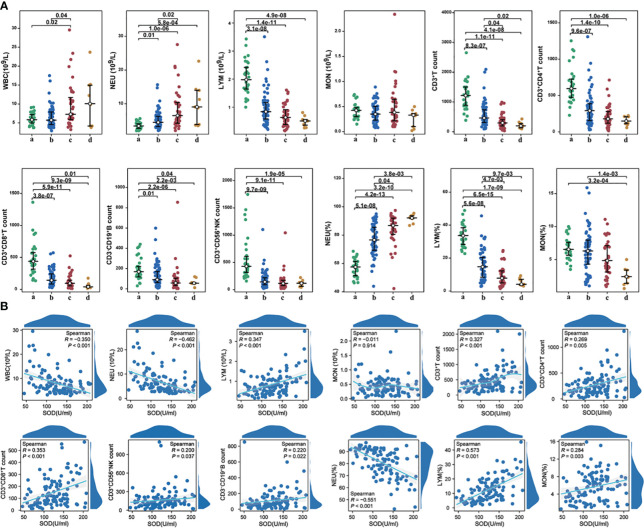
**(A)** The alterations in immunocytes among different groups; **(B)** The correlations between SOD and immunocytes (count or percentages) in COVID-19 cases. a, healthy controls; b, mild to moderate COVID-19; c, survival severe COVID-19; d, deceased severe COVID-19.

No significant differences were observed in WBC count between the mild to moderate group and the healthy controls. However, patients who developed severe disease exhibited significantly higher WBC counts. Comparative analysis of NEU count and its percentage demonstrated that both were significantly higher in the mild to moderate group and severe group compared to the control group. However, no significant difference was found between deceased severe subjects and surviving severe cases. LYM count and percentage were significantly lower in both the mild to moderate group and severe group, consistent with previous study (14). MON count did not differ significantly among the groups. Correlation analysis revealed significant negative correlations between SOD and WBC count (r=-0.350, P<0.001), NEU count (r=-0.462, P<0.001), and NEU percentage (r=-0.551, P<0.001), as well as significant positive correlations between SOD and LYM count (r=0.347, P<0.001) and LYM percentage (r=0.573, P<0.001).To investigate the immune response to the Omicron variant, flow cytometry was employed to analyze peripheral blood lymphocyte populations. A significant decrease in CD3^+^ T cell count was observed in the COVID-19 group. Moreover, CD3^+^ T cell count was notably lower in the severe group compared to the mild to moderate group. All other measured parameters, including CD3^+^CD4^+^ T cell count, CD3^+^CD8^+^ T cell count, CD3^-^CD19^+^ B cell count, and CD3^+^CD56^+^ NK cell count, were significantly lower in COVID-19 patients compared to the control group (**Figure 4A**). Correlation analysis revealed positive associations between SOD and CD3^+^ T cell count (r=0.327, P<0.001), CD3^+^CD4^+^ T cell count (r=0.269, P=0.005), CD3^+^CD8^+^ T cell count (r=0.353, P<0.001), CD3^-^CD19^+^ B cell count (r=0.220, P=0.022), and CD3^+^CD56^+^ NK cell count (r=0.200, P=0.037) (**Figure 4B**).

### Correlation of SOD with nutritional metabolic indicators

**Figure 5:**
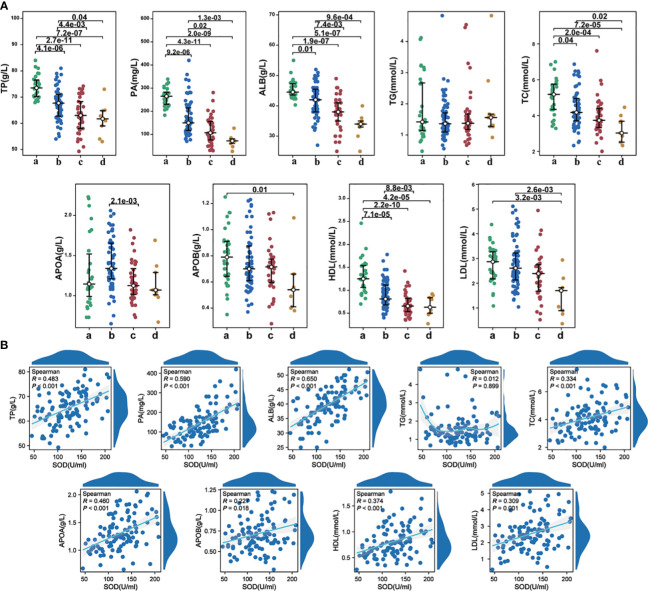
**(A)** The alterations in nutritional metabolic indicators among different groups; **(B)** The correlations between SOD and nutritional metabolic indicators in COVID-19 cases. a, healthy controls; b, mild to moderate COVID-19; c, survival severe COVID-19; d, deceased severe COVID-19.

We observed significantly reduced levels of TP, PA, and ALB in individuals with COVID-19 compared to the control group. Furthermore, these indicators exhibited even lower levels in the severe group as compared to the mild to moderate group (**Figure 5A**). Correlation analysis revealed notable positive associations between SOD and TP (r=0.483, P<0.001), PA (r=0.590, P<0.001) (**Figure 5B**), as well as ALB (r=0.650, P<0.001). Notably, we did not find any significant difference in TG levels among the four groups. HDL showed a significant decrease in COVID-19 patients, with considerably lower levels in the severe group than the mild to moderate group (**Figure 5A**). Correlation analysis demonstrated significant positive correlations between SOD and total TC (r=0.334, P<0.001), APOA (r=0.460, P<0.001), APOB (r=0.227, P=0.018), HDL (r=0.374, P<0.001), and LDL (r=0.309, P=0.001) (**Figure 5B**).

### Correlation of SOD with organ injury indexes

**Figure 6:**
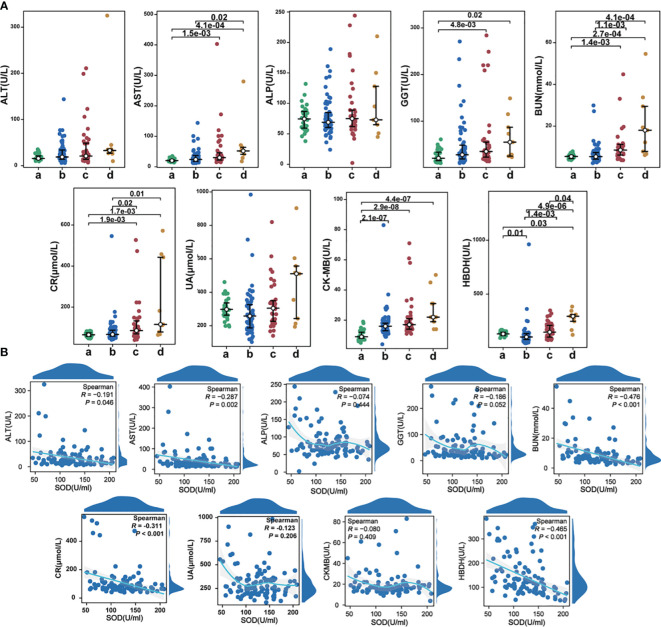
**(A)** The alterations in organ injury indexes among different groups; **(B)** The correlations between SOD and organ injury indexes in COVID-19 cases. a, healthy controls; b, mild to moderate COVID-19; c, survival severe COVID-19; d, deceased severe COVID-19.

Among the 63 patients in the mild to moderate group, 9 (24.3%) cases exhibited elevated ALT activity, 12 (19.0%) cases had elevated AST activity, 3 (4.8%) cases showed elevated ALP activity, and 19 (30.2%) cases presented elevated GGT activity. In the severe group, 9 (24.3%) cases displayed elevated ALT activity, 11 (29.7%) cases had elevated AST activity, 5 (13.5%) cases demonstrated elevated ALP activity, and 16 (43.2%) cases exhibited elevated GGT activity. Among the deceased severe group, 91 (11.1%) cases had elevated ALT activity, 7 (77.8%) cases showed elevated AST activity, 1 (11.1%) case had elevated ALP activity, and 5 (55.6%) cases presented elevated GGT activity (**Figure 6A**). The ratios of severe patients with elevated AST and GGT activity were significantly higher compared to the control group, indicating hepatocyte destruction and increased release of intracellular AST into the bloodstream in severe patients. Correlation analysis revealed significant negative correlations between SOD and ALT (r=-0.191, P=0.046) as well as AST (r=-0.287, P=0.002), but no significant correlation was observed with ALP and GGT (**Figure 6B**). These findings suggest that Omicron infection may also contribute to liver injury, as evidenced by changes in liver enzyme activity. Biomarkers of renal injury, such as BUN and CR, exhibited significant elevation in both mild to moderate and severe COVID-19 patients, with the highest levels observed in severe cases (**Figure 6A**). These findings suggest that Omicron infection may invade the kidney and impair renal function, particularly in severe cases. Significant negative correlations were observed between SOD and BUN (r=-0.476, P<0.001), as well as CR (r=-0.311, P<0.001) (**Figure 6B**). Moreover, myocardial enzymes, namely CKMB and HBDH, were demonstrated significantly higher levels in the disease groups, with the severe group exhibiting markedly higher levels than the mild to moderate group (**Figure 6A**). Correlation analysis revealed a significant negative correlation between SOD and HBDH (r=-0.465, P<0.001), but no significant correlation was found with CKMB (**Figure 6B**). These findings indicate that Omicron infection may lead to both renal and myocardial injury, as evidenced by elevated concentrations of related biomarkers.

### Correlation of SOD with coagulation indicators

**Figure 7:**
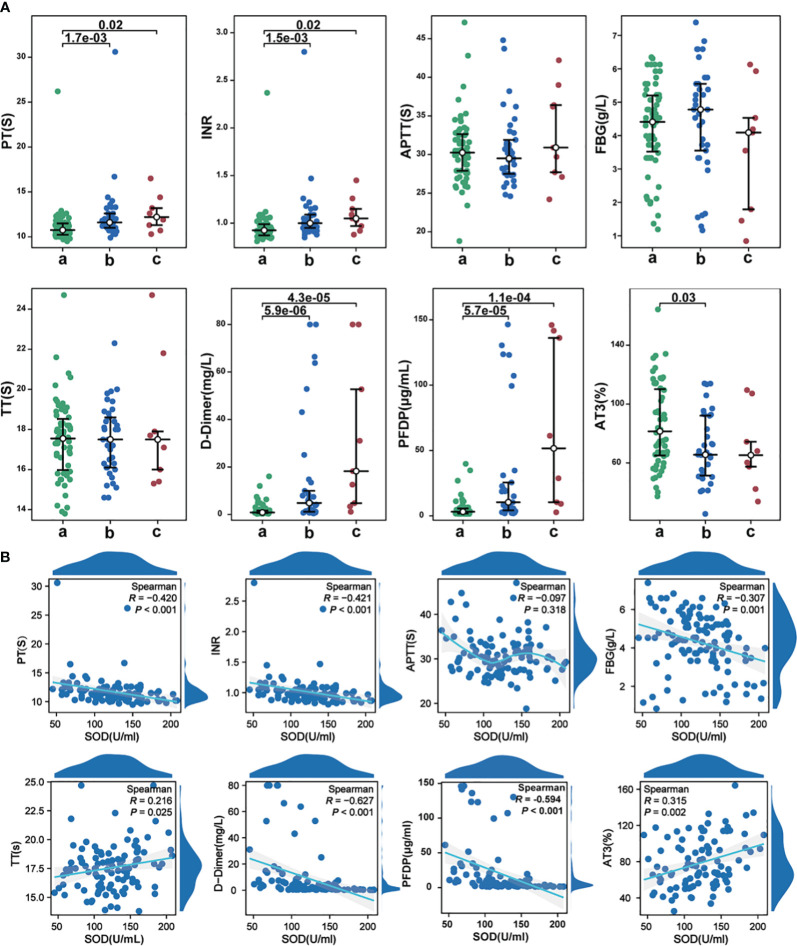
**(A)** The alterations in coagulation indicators among different groups; **(B)** The correlations between SOD and coagulation indicators in COVID-19 cases. a, mild to moderate COVID-19; b, survival severe COVID-19; c, deceased severe COVID-19.

In cases of severe COVID-19, levels of D-dimer and PFDP were significantly elevated compared to mild to moderate cases (**Figure 7A**). Our correlation analysis indicated that SOD exhibited associations with PT (r=-0.420, P<0.001), INR (r=-0.421, P<0.001), FBG (r=-0.275, P=0.004), D-dimer (r=-0.627, P<0.001), PFDP (r=-0.594, P<0.001), TT (r=0.216, P<0.025), and AT-III (r=0.315, P=0.002) (**Figure 7B**). However, no significant correlation was observed between SOD and APTT.

### ROC curves of SOD for identifying severe COVID-19 and predicting mortality

**Figure 8:**
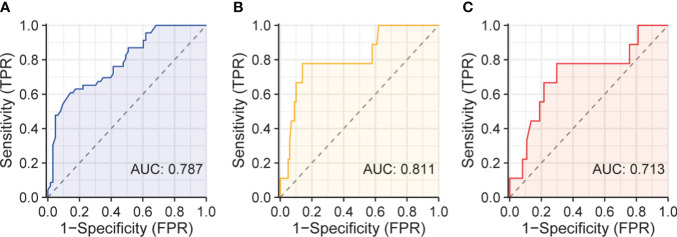
ROC curves of serum SOD. **(A)** ROC curve of serum SOD for identifying severe patients; **(B)** ROC curve for predicting death in all patients; **(C)** ROC curve for predicting death in severe patients.

In order to analyze the relationship between SOD and Omicron-infected COVID-19 severity and mortality, we draw ROC curves of SOD. The ROC curve in **Figure 8A** revealed an AUC of 0.787 for SOD in identifying severe cases. Furthermore, **Figure 8B** showed an AUC of 0.811 for SOD in prediction of mortality from all COVID-19 subjects. The last curve in **Figure 8C** explored an AUC of 0.713 for predicting mortality from severe COVID-19 subjects.

## Discussion

The current investigation has substantiated a noteworthy reduction in SOD activity within individuals afflicted by Omicron-infected COVID-19, closely aligning with systemic alterations. These findings propose that SOD might serve as a comprehensive and multifaceted proxy for organismal changes. Subgroup analysis has unveiled a substantial decline in SOD activity even among patients with mild to moderate COVID-19, aligning with prior researches (12, 13, 15). In fact, downregulation of SOD activity could be modulated via the JAK2/STAT1 signaling pathway, leading to the precipitation of pSTAT1 and SOD, as observed in mice immunized with the SARS-CoV-2 spike protein (16). Numerous viruses, including coronaviruses, elicit a biochemical imbalance in infected cells, thereby inducing oxidative stress, which could be linked to the development of pathologies and exacerbation of symptoms (17, 18). Hence, a lower level of SOD might indicate an unfavorable prognosis, underscoring the potential consideration of antioxidants as a prospective therapeutic approach against SARS-CoV-2 (19, 20). Nonetheless, conflicting outcomes have been reported in other studies. For instance, Sahar Golabi et al. observed significantly higher SOD activity in serum samples from new-onset COVID-19 patients across varying disease severities compared to healthy individuals (10). Similarly, Tavassolifar MJ et al. analyzed the impact of SARS-CoV-2 infection on intracellular and extracellular signaling gene expression via the redox system and noted a significant elevation in SOD1 and SOD2 gene levels within COVID-19 PBMCs (9). Additionally, a separate investigation in pregnant women with SARS-CoV-2 infection found no significant alterations in SOD activity among those with mild or asymptomatic COVID-19 in comparison to healthy pregnant women (21). Yaghoubi N et al. observed a declining trend in SOD activity among healthy individuals, mildly ill patients, and severely ill patients, although the difference was not statistically significant (22). Following a comparative analysis of similar studies, our findings align with the results obtained from the Chinese population (13) and Indian population (12), indicating that discrepancies in population demographics or characteristics of the infecting virus might contribute to the inconsistent outcomes.

Infection with SARS-CoV-2 disrupts the redox balance of intracellular and extracellular host immune cells, resulting in an imbalanced immune response and dysregulation of immune activation. This, in turn, leads to excessive inflammation in the lungs, contributing to the progression and exacerbation of the disease (9, 23, 24). Omicron-infected COVID-19 patients exhibited significantly elevated systemic inflammatory markers, including CRP, IL-6, LDH, and ferritin, which showed negative correlations with SOD activity. The inflammatory microenvironment is characterized by an abundance of macrophages and lymphocytes that generate high levels of ROS, feed-back influencing SOD activity by either depletion or compensation (25). This study further confirmed the imbalance of immune cells in patients, where individuals with severe illness displayed significantly increased WBC count, NEU count, and NEU percentage. The elevated immune cell counts were negatively correlated with SOD activity, while LYM count, LYM percentage, and LYM subsets such as CD3^+^CD4^+^ T cells, CD3^+^CD8^+^ T cells, CD3^-^CD19^+^ B cells, and CD3^+^CD56^+^ NK cells were all significantly reduced. These decreases in immune cell populations exhibited positive correlations with SOD. The symptoms of Omicron infection, particularly in elderly individuals, range from mild to severe, with some severe cases resulting in fatalities (26). The development of the infection is closely associated with oxidative stress, inflammation, and immune cell imbalances, triggering inflammation and stimulating the production of pro-inflammatory cytokines (25, 27). The immune cell imbalance is also evident in the impairment of CD4^+^ T cell function and the overactivation of CD8^+^ T cells in COVID-19, leading to a depletion of cell counts (14, 28). And the immune cells are energy intensive in their battle with the virus, increasing the body’s energy and nutritional needs. And the immune cells engage in high-energy consumption during their combat against the virus, thereby elevating the body’s energy and nutritional requirements (29). The life cycle of the virus is significantly influenced by lipid and cholesterol metabolism (30), and metabolic disorders afflict up to 50% of COVID-19 patients who succumb to the disease (31). COVID-19 cases exhibited substantially lower levels of TP, PA, and ALB, with the most severe patients displaying the lowest levels of these proteins. Correlation analysis demonstrated a positive association between SOD and TP, PA, and ALB. Moreover, severe patients also exhibited significant reductions in TC, APOA, APOB, and HDL. The diminished levels of lipid-related metabolism could be attributed to the absence of metabolic enzymes encoded by the SARS-CoV-2 genome that are necessary for viral genome replication, protein synthesis, and lipogenesis (32). As a consequence, the virus resorts to exploiting the metabolic networks and biogenic programs of host cells for self-replication, consequently leading to a decrease in host cell-associated proteins and lipids (33). SOD was observed to be positively correlated with TC, APOA, APOB, HDL, and LDL. The process of self-replication employed by SARS-CoV-2 utilizing the host cell’s lipid metabolic network generates a substantial quantity of free radicals, potentially necessitating the depletion of SOD for scavenging purposes, thereby resulting in reduced SOD activity (34). Nutritional status has the capacity to modulate oxidative stress, inflammation, and immune function, and it has been proposed that the host’s nutritional status plays a pivotal role in determining the outcome of various infectious diseases. Supplementation with micronutrients also represents an effective intervention for regulating oxidative stress and immune status in infected patients (35). Respiratory symptoms are predominant in COVID-19 patients, but evidence shows that some patients, especially those with severe disease, can develop organ damage, which may significantly increase mortality (13, 36, 37). Organ damage varies between sub-strains as the virus mutates (38). In our research, the biomarkers of renal damage, BUN and CR, were significantly elevated in all patients, even in mild to moderate cases. Additionally, activities of AST, GGT, CKMB, and HBDH were also significantly increased in severe cases, indicating different degrees of damage in liver, kidney, and myocardium. We found SOD was correlated with these enzymes. SOD can control the levels of various reactive oxygen species and reactive nitrogen species, limiting their potential toxicity (39), while reduced SOD activity leads to a decrease in mitochondrial antioxidant capacity, membrane lipid peroxidation and membrane lipid peroxidation, leading to increased levels of oxidative damage such as protein carbonylation and DNA breakage (40), which may influence mitochondrial function to compromise overall cellular health, thus cause damage to organs (41). Decreased SOD activity can also cause platelet damage and apoptosis, and the interaction of dysfunctional platelets with the coagulation cascade may exacerbate coagulation events (42). Indeed, we observed that decreased SOD activity was closely associated with indicators of coagulation abnormalities.

Moreover, the occurrence of long COVID after acute phase has attracted widespread attention, but there is currently a lack of early predictive biomarkers for its onset. Past researches have indicated a close association between the occurrence of complications in long COVID, including neurological onset, and the severity of acute illness and vascular endothelial dysfunction (43–45). Given that SOD can significantly improve vascular endothelial dysfunction (46, 47) and our study observed a substantial decrease in SOD among severe patients, we hypothesize that individuals with long COVID might experience a noticeable decline in SOD early in the course of the disease.

Here, we highlight SOD as a biomarker to assess the severity and prognosis of COVID-19. However, our study has some limitations, such as the small sample size and the limited geographical origin of the patients. Secondly, due to the reduced lethality of COVID-19 triggered by Omicron variant infection, the small number of patients who ultimately died at follow-up in this study may have compromised the statistical significance in assessing the value of SOD for predicting mortality, and future studies should include more multicenter populations for evaluation. Third, due to the nature of this study, we were unable to establish a causal relationship between changes in serum SOD activity and systematic changes in COVID-19. Finally, this study was based on the patients’ initial laboratory indicators at the time of hospitalization, rather than the patients’ results at the time of symptom presentation, which may allow for heterogeneity between patients. To our knowledge, this is also the first study to investigate changes in SOD in COVID-19 patients infected with the Omicron variant, adding to the body of knowledge in this area.

## Data availability statement

The original contributions presented in the study are included in the article/supplementary material. Further inquiries can be directed to the corresponding author.

## Ethics statement

The studies involving humans were approved by Ethics Committee of Jiujiang NO.1 People’s Hospital. The studies were conducted in accordance with the local legislation and institutional requirements. The ethics committee/institutional review board waived the requirement of written informed consent for participation from the participants or the participants’ legal guardians/next of kin because The study was retrospective and did not require informed consent from patients.

## Author contributions

GX: Conceptualization, Funding acquisition, Investigation, Project administration, Supervision, Validation, Writing – review & editing. JC: Data curation, Investigation, Methodology, Software, Visualization, Writing – original draft. LH: Conceptualization, Data curation, Formal analysis, Investigation, Methodology, Software, Visualization, Writing – original draft. XL: Investigation, Resources, Software, Supervision, Writing – original draft. HX: Data curation, Formal analysis, Resources, Validation, Writing – original draft. FJ: Data curation, Investigation, Methodology, Software, Writing – original draft. WZ: Data curation, Investigation, Methodology, Software, Writing – original draft. LW: Data curation, Investigation, Project administration, Writing – original draft.
